# Increased expression of osteopontin in subchondral bone promotes bone turnover and remodeling, and accelerates the progression of OA in a mouse model

**DOI:** 10.18632/aging.203707

**Published:** 2022-01-04

**Authors:** Chuangxin Lin, Zhong Chen, Dong Guo, Laixi Zhou, Sipeng Lin, Changchuan Li, Shixun Li, Xinjia Wang, Bendan Lin, Yue Ding

**Affiliations:** 1 Department of Orthopedics, Sun Yat-Sen Memorial Hospital, Sun Yat-Sen University, Guangzhou 510120, P.R. China; 2 Department of Orthopedic Surgery, Shantou Central Hospital, Shantou 515000, P.R. China; 3 Department of Joint Surgery, Center for Orthopedic Surgery, The Third Affiliated Hospital of Southern Medical University, Guangzhou 510515, P.R China; 4 Department of Orthopedic, Affiliated Cancer Hospital, Shantou University Medical College, Shantou 515041, P.R. China

**Keywords:** OA, subchondral bone, osteopontin, bone turnover, remodeling

## Abstract

Osteopontin (OPN) has been proved to be closely related to the pathogenesis of osteoarthritis (OA), but the role of OPN in the pathogenesis of OA has not been fully clarified. Current studies on OPN in OA mostly focus on articular cartilage, synovial membrane and articular fluid, while ignoring its role in OA subchondral bone turnover and remodeling. In this study, we used a destabilization OA mouse model to investigate the role of OPN in OA subchondral bone changes. Our results indicate that increased expression of OPN accelerates the turnover and remodeling of OA subchondral bone, promotes the formation of h-type vessels in subchondral bone, and mediates articular cartilage degeneration induced by subchondral bone metabolism. In addition, our results confirmed that inhibition of PI3K/AKT signaling pathway inhibits OPN-mediated OA subchondral bone remodeling and cartilage degeneration. This study revealed the role and mechanism of OPN in OA subchondral bone, which is of great significance for exploring specific biological indicators for early diagnosis of OA and monitoring disease progression, as well as for developing drugs to regulate the metabolism and turnover of subchondral bone and alleviate the subchondral bone sclerosis of OA.

## INTRODUCTION

Osteoarthritis (OA) is a degenerative disease caused by obesity, aging, injury, heredity and other factors, which often occurs in the elderly. According to research reports, by the end of 2020, OA affected 7% of the world’s population, more than half a billion people, with women particularly affected [1]. At present, the commonly used prevention and treatment methods for OA are mainly the application of non-steroidal anti-inflammatory drugs (NSAIDs), or cartilaginous nutrition and protective drugs, such as glucosamine [2, 3]. However, long-term use of NSAIDs causes a variety of side effects affecting the gastrointestinal, renal and cardiovascular systems [4], and these treatments are limited to symptom control and remission progression, resulting in uncertain treatment outcomes in some patients with OA [5, 6]. For patients with end-stage OA and persistent severe pain, joint replacement is the only option. Therefore, it would be more beneficial to focus on the early stages of OA and to prevent OA. However, the biggest difficulty in early diagnosis of OA is the lack of specific and sensitive biological indicators to help clinical evaluation and monitoring of disease progression, and the lack of effective early treatment drugs. Therefore, early identification and prevention of OA is the key to improve the treatment effect.

Osteopontin (OPN) is a negatively charged non-collagenous bone matrix glycoprotein, which is widely distributed in extracellular matrix, bone tissue and inflammatory sites. As a cytokine, OPN acts on cell surface receptors such as integrins and CD44, and is involved in the regulation of inflammation, immunity, bone metabolism and other physiological and pathological processes [7]. ICOSL has recently been described as a novel OPN ligand expressed in osteoclasts and endothelial cells [8, 9, 10]. Interestingly, ICOSL triggering through its classic ligand ICOS generally induces opposite effects than those induced by its triggering through OPN [9, 11]. A large number of studies have shown that OPN is closely related to OA [12]. In cartilage of OA patients and individuals with joint damage, OPN mRNA expression is increased. OPN promotes chondrocyte proliferation [13, 14] and apoptosis [15], and up-regulates the expression levels of MMP13 [16], interleukin 6 (IL-6) and interleukin 8 (IL-8) in human OA chondrocytes [17], which may be one of the potential mechanisms of OPN participating in the pathogenesis of OA. In articular cartilage, the correlation between Wnt5a and OPN may be of great significance to the occurrence and pathogenesis of OA [18]. In addition, OPN mediates synovial hyperplasia and inflammation to induce OA [19, 20], which is also an important mechanism of OA pathogenesis. The levels of OPN in the serum and synovial fluid of patients with OA has been shown to be significantly increased, and to be positively correlated with the severity of OA [21].

However, the role of OPN in the pathogenesis of OA has not been fully elucidated, and most studies on OPN in OA focus on articular cartilage, synovial membrane, and articular fluid, while ignoring its role in subchondral bone turnover. Cartilage is closely related to subchondral bone. Cartilage damage and degeneration are inseparable from the structural destruction of subchondral bone, so cartilage and subchondral bone often act together as a complete functional unit [22]. The subchondral bone below the articular cartilage protects the articular cartilage and absorbs stress from the articular cartilage [22, 23], and it also has frequent molecular and signal communication with cartilage [24]. In this study, we found that OPN expression was increased not only in OA cartilage and synovium, but also in OA subchondral bone, and we explored the pathological significance and regulatory mechanism of the increased expression of OPN in OA subchondral bone turnover and remodeling, which may provide a potential target for early diagnosis, treatment and drug development of OA.

## RESULTS

### The expression of secreted osteopontin (sOPN) is increased in subchondral bone in OA

**Figure 1:**
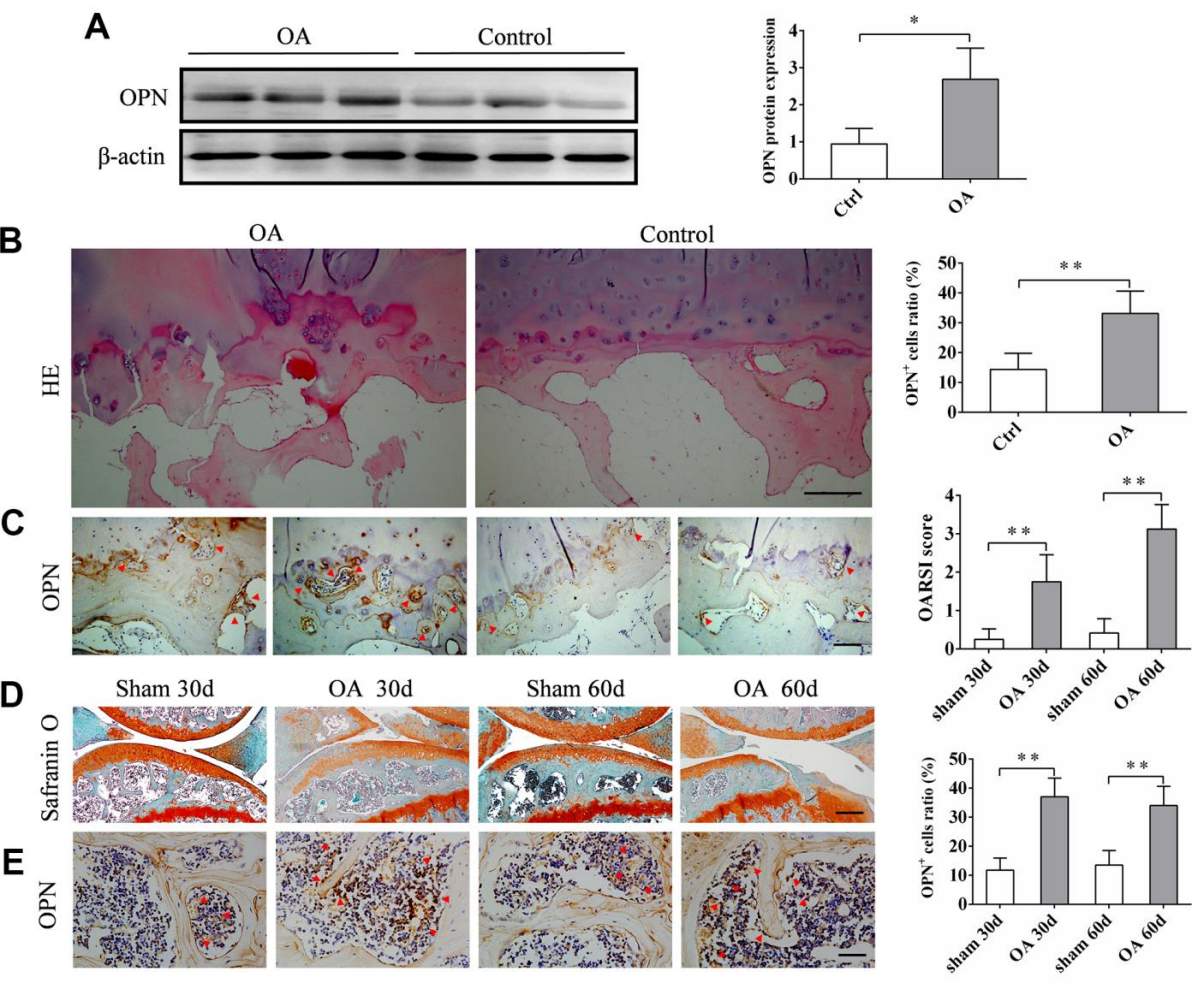
**The expression of sOPN is increased in subchondral bone in OA.** (**A**) Western blot analysis and quantification of the expression of sOPN in subchondral bone of patients with OA and control subjects. (**B**, **C**) Representative H&E, immunostaining and quantitative analysis of sOPN+ cells in the tibial subchondral bone of OA patients and control subjects. Positive cells were indicated with arrows. Scale bars = 200 μm (**B**), 100 μm (**C**), n ≥5. (**D**) Representative safranin O-fast green staining and OARSI scores of an OA mouse model and control group. Scale bars = 200 μm, n = 6. (**E**) Immunostaining and quantitative analysis of sOPN+ cells in the tibial subchondral bone of an OA mouse model and control group. Positive cells were indicated with arrows. Scale bars =50 μm, n = 6. Data are shown as mean ± s. d. and were analyzed by Student’s t test; *P < 0.05,**P < 0.01.

**Figure 2:**
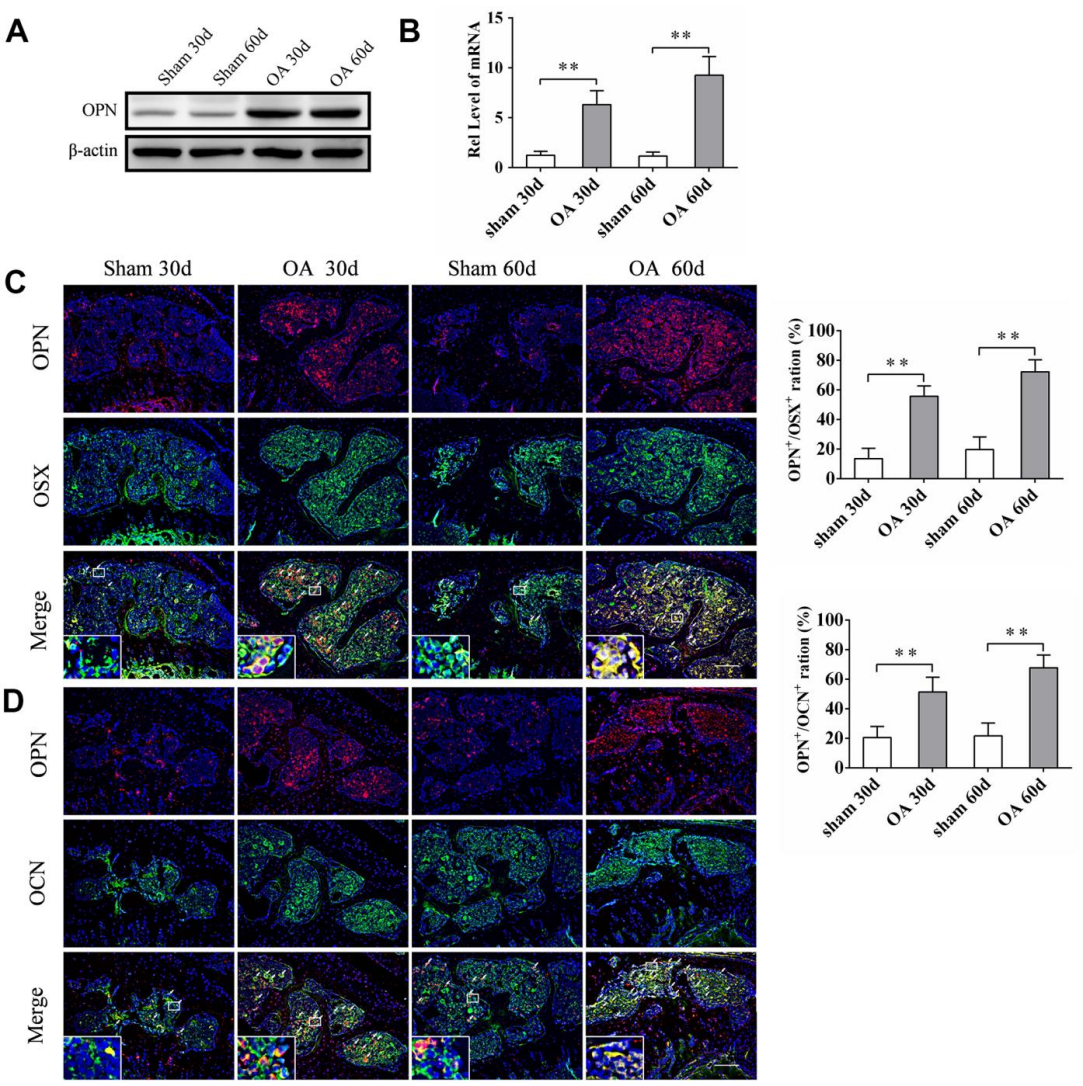
**sOPN was increased expressed in OA subchondral bone mainly in preosteoblasts and osteoblasts.** (**A**, **B**) Western blot and qPCR analysis of the expression of OPN in primary osteoblasts of subchondral bone of an OA mouse model and control group; n ≥ 3. (**C**, **D**) Representative immunofluorescent and quantitative analysis of sOPN in OSX+ preosteoblasts or OCN+ osteoblasts in the tibial subchondral bone of an OA mouse model and control group. Positive cells were indicated with arrows. The boxed area is magnified in the corner, scale bars = 50 μm. Data are shown as mean ± s. d. and were analyzed by Student’s t test; n = 6, *P < 0.05,**P < 0.01.

The expression of sOPN in subchondral bone tissue samples of OA patients and control subjects was analyzed by western blotting. The results showed that the expression of sOPN was significantly higher in OA patients compared to control subjects (Figure 1A). HE and immunohistochemical staining also showed that sOPN-positive cells in the subchondral bone of OA patients were significantly more than those in the control group (Figure 1B, 1C). We established an OA mouse model induced by anterior cruciate ligament transection and destabilized the medial meniscus (ACLT + DMM) and analyze the expression of sOPN in the subchondral bone tissues of OA mice and control mice at 30 or 60 days after operation. As expected, histochemical staining confirmed the presence of significantly more sOPN-positive cells in the subchondral bone of the OA model mice at 30 and 60 days after surgery than in the corresponding sham group (Figure 1D, 1E). The results of western blotting and real-time quantitative PCR also showed that the expression levels of sOPN protein and mRNA in primary osteoblasts of the subchondral bone tissue of OA mice were significantly higher than those in the corresponding sham group at 30 and 60 days after ACLT + DMM modeling (Figure 2A, 2B). In order to further determine the phenotype of sOPN-positive cells in subchondral bone, we performed double immunofluorescence staining on mouse subchondral bone tissue samples. Immunofluorescence staining showed that sOPN-positive cells in subchondral bone tissue of OA mouse model were mainly osterix (OSX, a transcriptional activator essential for preosteoblast differentiation)-positive cells (Figure 2C) and osteocalcin (OCN, produced by osteoblasts and generally regarded as a marker of bone formation) -positive cells (Figure 2D).

### OPN regulates OA subchondral bone metabolism and remodeling

**Figure 3:**
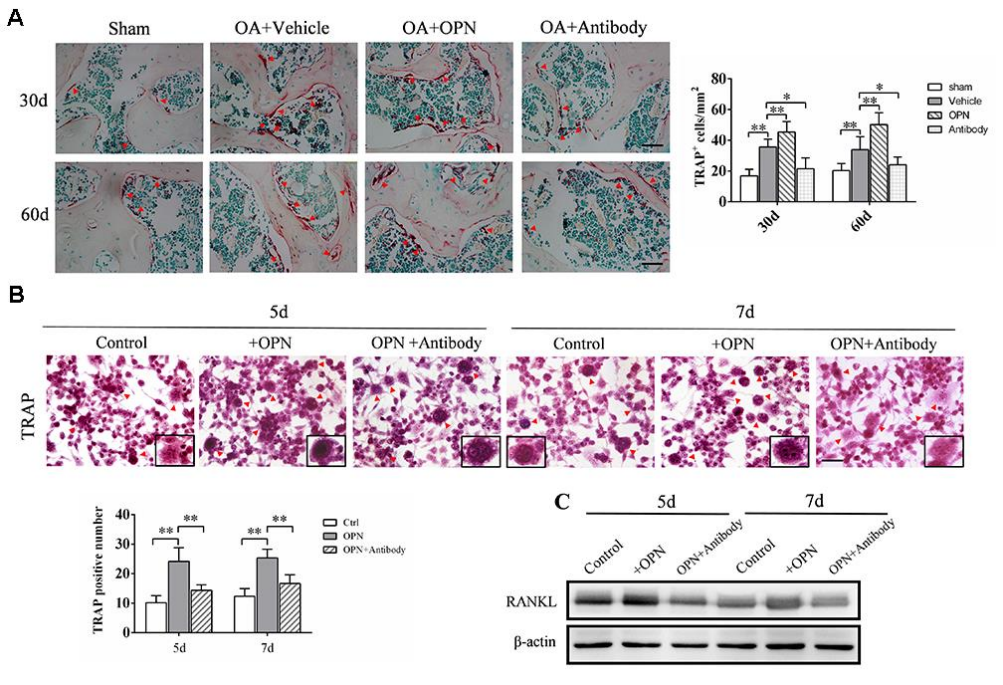
**OPN promotes osteoclastogenesis in the subchondral bone in OA.** (**A**) TRAP staining and quantitative analysis of osteoclasts in the tibial subchondral bone of an OA mouse model treated with vehicle, rmOPN or neutralizing antibody, and sham group. Positive cells were indicated with arrows; scale bars = 50 μm, n = 6. (**B**) TRAP staining of BMMs treated with M-CSF (20 ng/ml) and RANKL (50 ng/ml) followed by the stimulation with rmOPN (100 ng/mL) and antibody (1.0 μg/mL) for 5 or 7 days. Positive cells were indicated with arrows. TRAP-positive multinuclear cells containing more than three nuclei were counted as osteoclasts; scale bars = 50 μm, n = 6. (**C**) Western blot analysis of the expression of RANKL in BMMs treated with M-CSF and RANKL followed by the stimulation with rmOPN (100 ng/mL) and antibody (1.0 μg/mL) for 5 or 7 days. Data are shown as mean ± s. d and were analyzed by one-way ANOVA; *P < 0.05, **P < 0.01.

Since we found that the number of Tartrate-resistant acid phosphatase (TRAP)-positive cells (osteoclasts) in the subchondral bone of OA mice was significantly higher than that of the corresponding sham group at 30 and 60 days after the model was established (Figure 3A), and previous studies including ours [25] also confirmed that the number of osteoclasts in the subchondral bone of OA mice was significantly increased at the early stage of osteoarthritis. Therefore, we examined the effect of exogenous OPN or OPN neutralizing antibody treatment on the number of osteoclasts in subchondral bone. After OA model was established, recombinant mouse OPN (rmOPN, 200 μg/mouse) or OPN neutralizing antibody (50 μg/mouse) was injected intraperitoneally twice a week for 4 or 8 weeks. The control groups were treated with saline as vehicle for the same periods. The number of TRAP-positive cells in the subchondral bone of OA mice treated with rmOPN was significantly increased than that of subchondral bone in the OA+vehicle group (Figure 3A). While the number of TRAP-positive cells in the subchondral bone of OA mice treated with OPN neutralizing antibody was significantly reduced than that of subchondral bone in the OA+vehicle group (Figure 3A). In addition, we isolated and extracted mouse bone marrow macrophages (BMMs) for *in vitro* experiments. BMMs were differentiated into osteoclasts by RANKL and M-CSF, and the effect of OPN on osteoclast differentiation was verified by adding rmOPN (100 ng /mL) and OPN neutralizing antibody (1.0 μg/mL). TRAP staining was performed after 5 or 7 days of stimulation. TRAP staining results showed that the number of TRAP-positive cells in BMMs added with rmOPN was significantly higher than that in the control group (Figure 3B). However, BMMs supplemented with rmOPN and OPN neutralizing antibody differentiated significantly fewer TRAP-positive cells than those supplemented with rmOPN (Figure 3B). Western blot analysis further showed that the addition of rmOPN promoted the expression of receptor activator of nuclear factor kappa-B ligand (RANKL, a member of the TNF family, triggers osteoclastogenesis by forming a complex with RANK) in BMMs cultured *in vitro* (Figure 3C). These results also suggest that OPN is involved in inducing osteoclast differentiation in OA subchondral bone.

**Figure 4:**
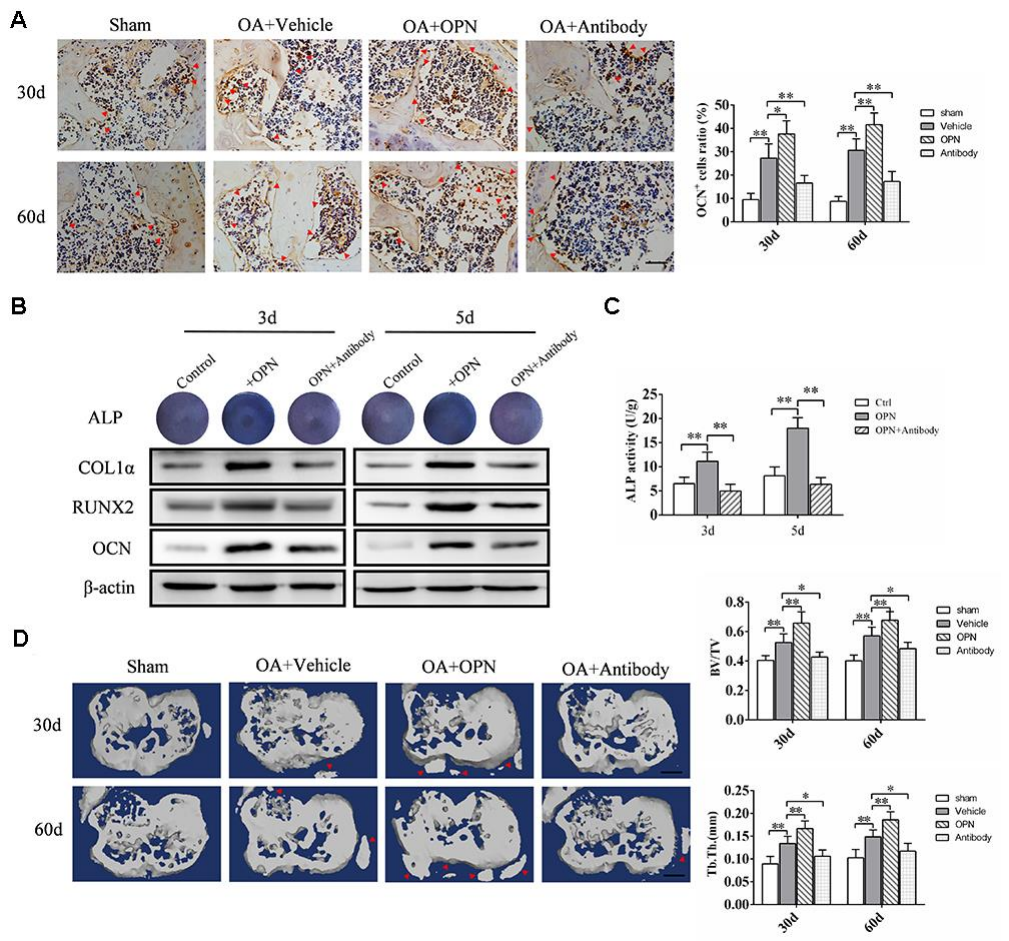
**OPN regulates OA subchondral bone metabolism and accelerates subchondral bone remodeling.** (**A**) Immunostaining of OCN+ cells in the tibial subchondral bone of an OA mouse model treated with vehicle, rmOPN or neutralizing antibody, and sham group. Positive cells were indicated with arrows, scale bars = 100 μm, n=6. (**B**) ALP staining and western blot analysis of collagen 1α (COL1α), RUNX2, and OCN expression of MC3T3-E1 cells treated with Dex (10^−7^ M), β-glycerophosphate (10 mM) and VC (50 μg/ml) followed by the stimulation with rmOPN and OPN antibody for 3 or 5 days. (**C**) ALP activity of MC3T3-E1 cells treated with Dex (10^−7^ M), β-glycerophosphate (10 mM) and VC (50 μg/ml) followed by the stimulation with rmOPN and antibody for 3 or 5 days, n=6. (**D**) Representative 3D reconstruction of micro-CT images of the tibial subchondral bone of an OA mouse model treated with vehicle, rmOPN or antibody, and sham group. Osteophyte were indicated with arrows, scale bar = 1 mm. Quantitative analysis of bone volume/total volume (BV/TV) and trabecular thickness (Tb. Th.), n=6. Data are shown as mean ± s. d. and were analyzed by one-way ANOVA, *P < 0.05, **P < 0.01.

The above experimental results suggested that the increased expression of sOPN in OA subchondral bone mainly came from preosteoblasts and osteoblasts. And immunohistochemical staining showed the number of OCN-positive cells (osteoblasts) in OA mice subchondral bone at 30 and 60 days after modeling was significantly higher than that in the corresponding control group (Figure 4A). Therefore, we also analyzed the effect of exogenous OPN or OPN neutralizing antibody treatment on the osteoblasts in subchondral bone. The number of OCN-positive cells in the subchondral bone of OA mice treated with rmOPN was significantly increased than that of subchondral bone in the OA+vehicle group (Figure 4A). OCN-positive cells in the subchondral bone of OA mice treated with OPN neutralizing antibody was significantly decreased than that of subchondral bone in the OA+vehicle group (Figure 4A). In addition, we used MC3T3-E1 cells for *in vitro* experiments. MC3T3-E1 cells were induced to differentiate into osteoblasts by osteoblast induction medium containing Dex, β-glycerophosphate and VC, and the effect of OPN on osteoblast differentiation was verified by adding rmOPN (100 ng /mL) and OPN neutralizing antibody (1.0 μg/mL). Western blotting was performed 3 or 5 days after induction and showed that the expression of collagen 1α, RUNX family transcription factor 2 (RUNX2) and OCN in MC3T3-E1cells added with rmOPN were significantly higher than those in the control group (Figure 4B). Collagen 1α, RUNX2 and OCN in MC3T3-E1cells added with rmOPN and OPN-neutralizing antibody were significantly lower than those added with rmOPN (Figure 4B). Alkaline phosphatase (ALP) staining and ALP activity assay showed that the ALP staining and ALP activity of MC3T3-E1cells treated with rmOPN were significantly higher than those in the control group at 3 and 5 days after osteogenic induction (Figure 4C). In contrast, MC3T3-E1 cells treated with rmOPN and OPN neutralizing antibody showed significantly lower ALP staining and ALP activity than those treated with rmOPN (Figure 4C).

Furthermore, micro-CT scan analysis showed significant changes in the morphology and structure of subchondral bone in mice at 30 and 60 days after OA modeling (Figure 4D). Intraperitoneal injection of rmOPN accelerated subchondral bone turnover and remodeling in OA mice, resulting in further destruction of subchondral bone morphology and structure, and osteophyte formation at the joint edge (Figure 4D). Injection of OPN neutralizing antibody alleviated bone remodeling in OA mice and protected normal subchondral bone morphology (Figure 4D). These results further suggest that OPN is involved in the regulation of OA subchondral bone metabolism and bone remodeling.

### OPN promotes the formation of h-type vessels in subchondral bone in OA

**Figure 5:**
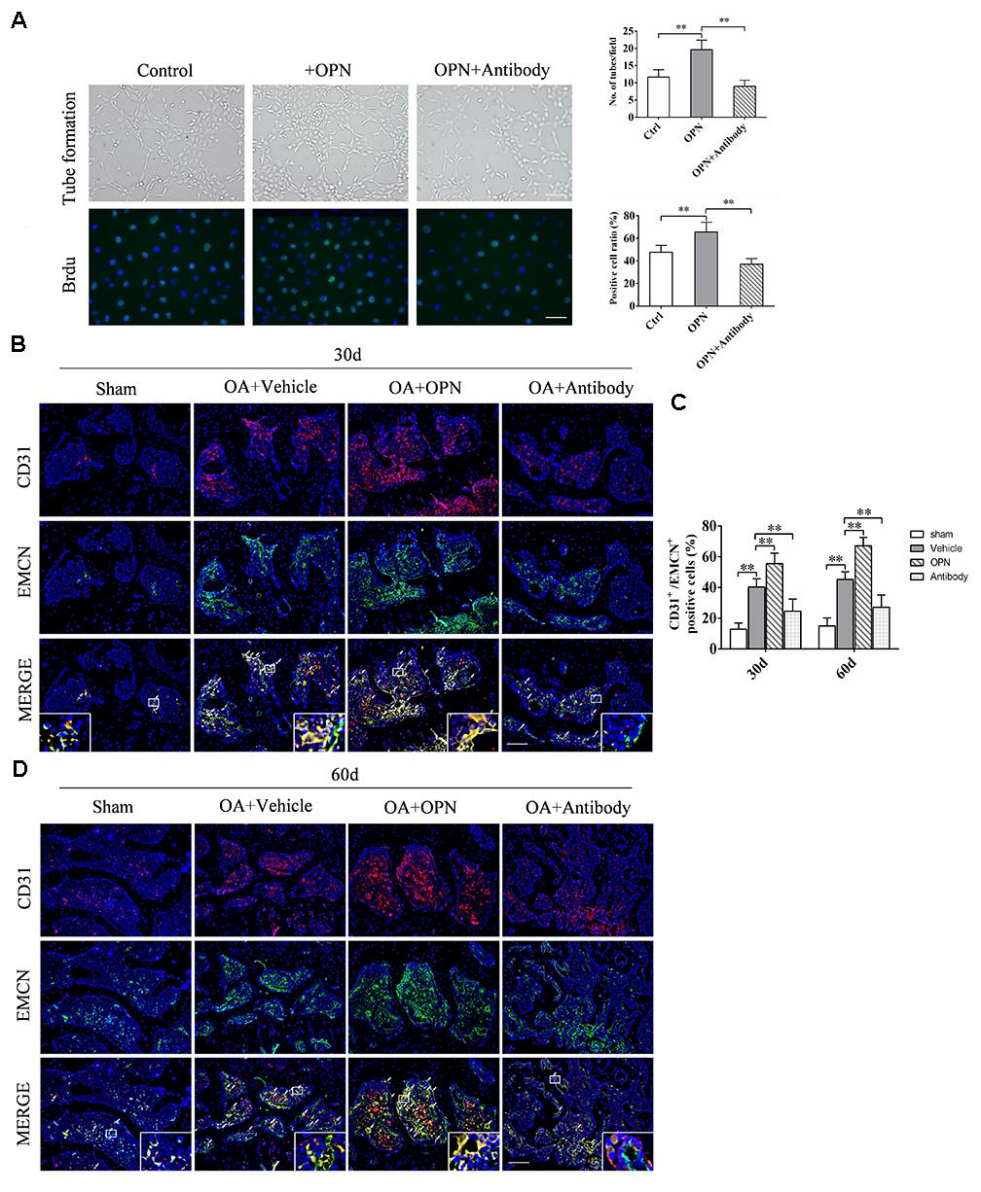
**OPN promotes the formation of h-type vessels in subchondral bone of OA.** (**A**) HUVECs were treated with rmOPN (100 ng/mL) and neutralizing antibody (1.0 μg/mL) for 24 h, and tubes were measured using a tube formation assay; scale bars = 100 μm, n = 6. (**B**) Representative images and quantitative analysis of Brdu (green) immunofluorescence in HUVECs treated with rmOPN (100 ng/mL) and neutralizing antibody (1.0 μg/mL) for 24 h; scale bars = 25 μm, n = 6. (**C**, **D**) Representative images and quantitative analysis of CD31 and Endomucin (EMCN) co-immunostaining in tibial subchondral bone of an OA mouse model treated with vehicle, rmOPN or neutralizing antibody, and sham group. Positive cells were indicated with arrows. Boxed area is magnified in the corner. Scale bars = 50 μm. Data are shown as mean ± s. d. and were analyzed by one-way ANOVA, n = 6, *P < 0.05, **P < 0.01.

Bone metabolism is closely related to bone angiogenesis, especially h-type vessels. Therefore, the effect of OPN on OA subchondral bone angiogenesis was detected and verified by adding rmOPN or OPN neutralizing antibody to Human Umbilical Vein Endothelial Cells (HUVECs) *in vitro* culture. HUVECs were treated with rmOPN (100 ng /mL) and OPN neutralizing antibody (1.0 μg/mL) for 24h, and cultured on matrigel to induce tube formation, and then photographed under a light microscope. The results of tube forming experiment showed that the number of tubes formed by HUVECs added with rmOPN was significantly higher than that in the control group, while the number of tubes formed by HUVECs added with rmOPN and OPN neutralizing antibody was significantly lower than that added with rmOPN (Figure 5A). We further detected the effect of OPN on the proliferation of vascular endothelial cells by Brdu labeled HUVECs. The results showed that the ratio of Brdu-positive cells after rmOPN (100 ng /mL) stimulation for 24h was significantly higher than that in the control group, while the ratio of Brdu-positive cells after rmOPN and neutralizing antibody stimulation(1.0 μg/mL) for 24h was significantly lower than that added with rmOPN (Figure 5B). Moreover, we found the h-type vessels of subchondral bone in OA mouse model were significantly more than those in the corresponding sham group at 30 and 60 days (Figure 5C, 5D). After rmOPN or OPN neutralizing antibody treatment, the number of subchondral h-type vessels of OA mice treated with rmOPN was significantly increased than that in the OA+vehicle group at 30 and 60 days (Figure 5C, 5D); while the number of h-type vessels in subchondral bone treated with OPN neutralizing antibody was significantly reduced compared with that in the OA+ vehicle group at 30 and 60 days (Figure 5C, 5D). These results suggest that OPN promotes neovascularization in OA subchondral bone.

### OPN accelerates the degeneration of OA articular cartilage

**Figure 6:**
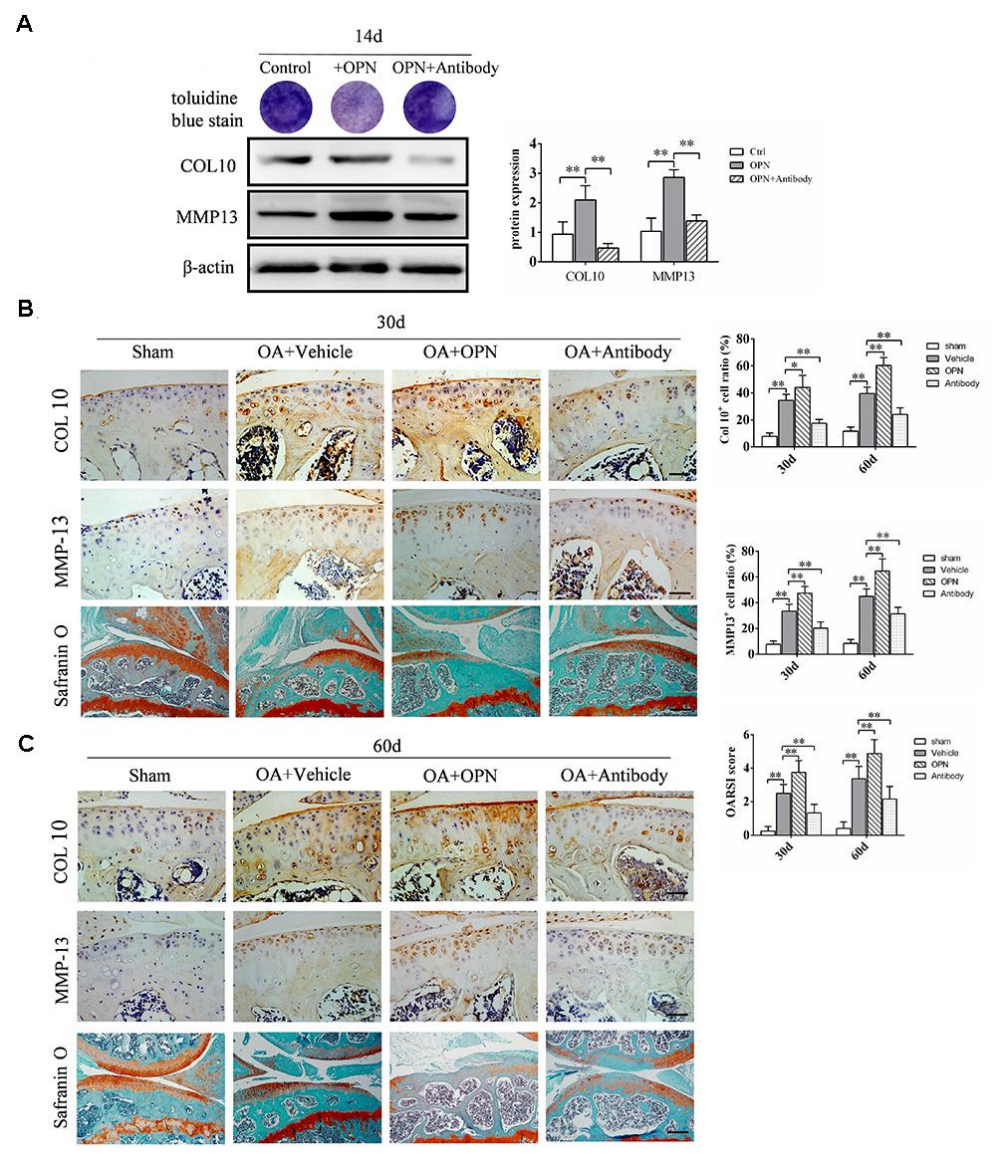
**OPN accelerates the degeneration of OA articular cartilage.** (**A**) Toluidine blue staining of ADTC5 cells treated with ITS (10 μg/mL) followed by stimulation with rmOPN (100 ng/mL) and neutralizing antibody (1.0 μg/mL) for 14 days. Western blot analysis and quantification of the expression of collagen-10 (COL10) and MMP-13 in ADTC5 cells treated with ITS followed by stimulation with rmOPN and neutralizing antibody for 14 days. (**B**, **C**) Representative immunostaining and quantitative analysis of COL10+, MMP-13+ cells in articular cartilage of an OA mouse model treated with vehicle, rmOPN or neutralizing antibody, and sham group. Scale bars = 50 μm. Safranin O-fast green staining and OARSI scores of tibial articular cartilage and subchondral bone of an OA mouse model treated with vehicle, rmOPN or neutralizing antibody, and sham group. Scale bars = 200 μm. Data are shown as mean ± s. d. and were analyzed by one-way ANOVA, n=6, *P < 0.05, **P < 0.01.

Since subchondral bone and articular cartilage can interact through cytokines and articular chondrocytes can express the OPN receptor CD44, we further investigated the effect of OPN on chondrocytes. We used ADTC5 cells for *in vitro* experiments. ADTC5 cells were induced to differentiate into mature chondrocytes by ITS, and the effect of OPN on chondrocyte differentiation was verified by adding rmOPN and OPN neutralizing antibody. Toluidine blue staining showed that the staining of ADTC5 cells after 14 days of stimulation with rmOPN was lighter than that treated with rmOPN, while the staining of ADTC5 cells treated with rmOPN and OPN neutralizing antibody was deeper than that treated with rmOPN (Figure 6A). It is generally accepted that metachromatic staining with toluidine blue represents cartilaginous matrix and that the degree of positive staining corresponds with the amount of proteoglycans. In addition, western blotting analysis showed that the expression of collagen 10 and matrix metallopeptidase 13(MMP13) in ADTC5 cells treated with rmOPN for 14 days were higher than those in the control group, while the expression of collagen 10 and MMP13 in ADTC5 cells treated with OPN neutralizing antibody for 14 days were lower than those treated with rmOPN (Figure 6A). Immunohistochemical results showed that the ratio of collagen 10- and MMP13-positive cells in cartilage tissues of OA mice intraperitoneally injected with rmOPN was significantly higher than that of OA mice intraperitoneally injected with vehicle at 30 and 60 days. However, the ratio of collagen 10- and MMP13-positive cells in cartilage of mice injected with OPN neutralizing antibody was significantly lower than that of OA mice injected with vehicle at 30 and 60 days (Figure 6B, 6C). Safranine O-fast green staining showed that the degeneration of articular cartilage in OA mice injected with rmOPN was significantly more serious than that in OA mice injected with vehicle at 30 and 60 days, and the degeneration of articular cartilage in OA mice injected with OPN neutralizing antibody was significantly reduced than that in OA mice injected with vehicle at 30 and 60 days (Figure 6B, 6C). These data suggest that the expression of OPN promotes the catabolism of articular cartilage in OA.

### Inhibition of the PI3K/AKT signaling pathway inhibits OPN-mediated subchondral bone and cartilage degeneration in OA

Previous studies have shown that the expression of OPN may be closely related to the activity of PI3K/ AKT signaling pathway [13, 24]. We verified this by knocking out PTEN, an inhibitory molecule upstream of PI3K/AKT signaling pathway, in MC3T3-E1 cells transfected with siRNA *in vitro*. The expression of p-AKT and OPN increased 48 h after transfection with PTEN-siRNA (Figure 7A). The addition of LY294002, a specific inhibitor of PI3K/AKT signaling pathway, inhibited the expression of p-AKT (Figure 7A), and the expression of OPN decreased accordingly (Figure 7A). In addition, we found significantly more OPN and p-AKT double positive cells in the subchondral bone of OA mice than in the corresponding sham group. Intraperitoneal injection of LY294002 could significantly inhibit the increase of OPN and p-AKT double positive cells in the subchondral bone of OA mice (Figure 7B). In addition, immunohistochemical staining showed that LY294002 injection could inhibit the pathological characteristics of abnormal increase of subchondral TRAP-positive cells (osteoclasts) and OCN-positive cells (osteoblasts) in OA mice (Figure7C). Furthermore, LY294002 injection inhibited the increase in the number of collagen 10-and MMP13-positive cells in the articular cartilage of mice after OA modeling (Figure 7C). Micro-CT scan showed that LY294002 injection reduced subchondral bone remodeling and structural damage in OA mice (Figure 7C). Safranine O-fast green staining showed that LY294002 injection reduced articular cartilage degeneration in OA mice (Figure 7C). These results suggest that inhibition of PI3K/AKT signaling can inhibit OPN-mediated OA subchondral bone and cartilage degeneration.

**Figure 7:**
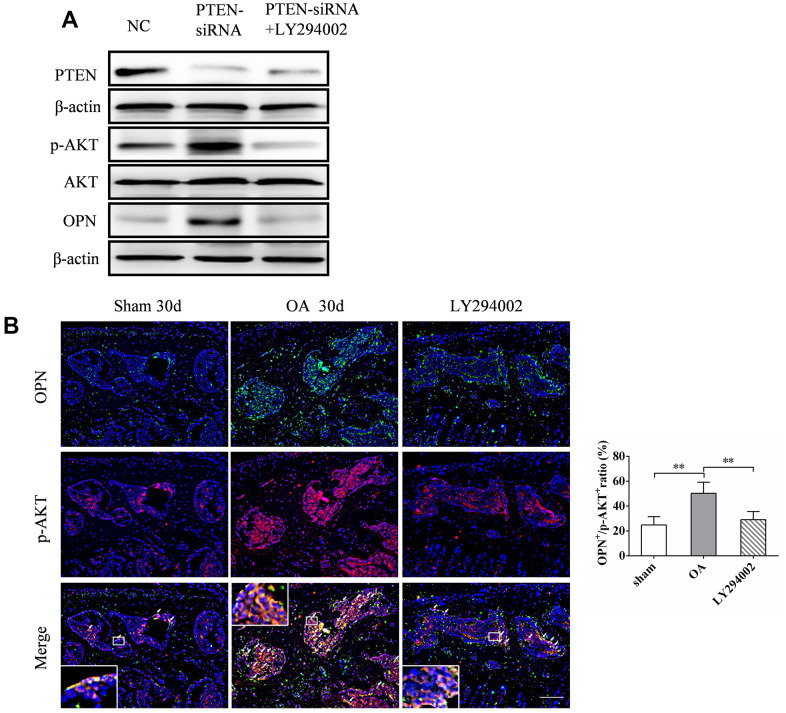
**LY294002 inhibits OPN-mediated subchondral bone and cartilage degeneration in OA.** (**A**) Western blot analysis of the expression of PTEN, p-AKT and OPN in MC3T3-E1 cells transfected with PTEN-siRNA and treated with LY294002 (10 μM) for 48 h. (**B**) Representative images and quantitative analysis of p-AKT and OPN co-immunostaining in the tibial subchondral bone in sham group, OA group and LY294002 treatment group. Positive cells were indicated with arrows. Boxed area is magnified in the corner. Scale bars = 50 μm, n = 6.

**Figure 7:**
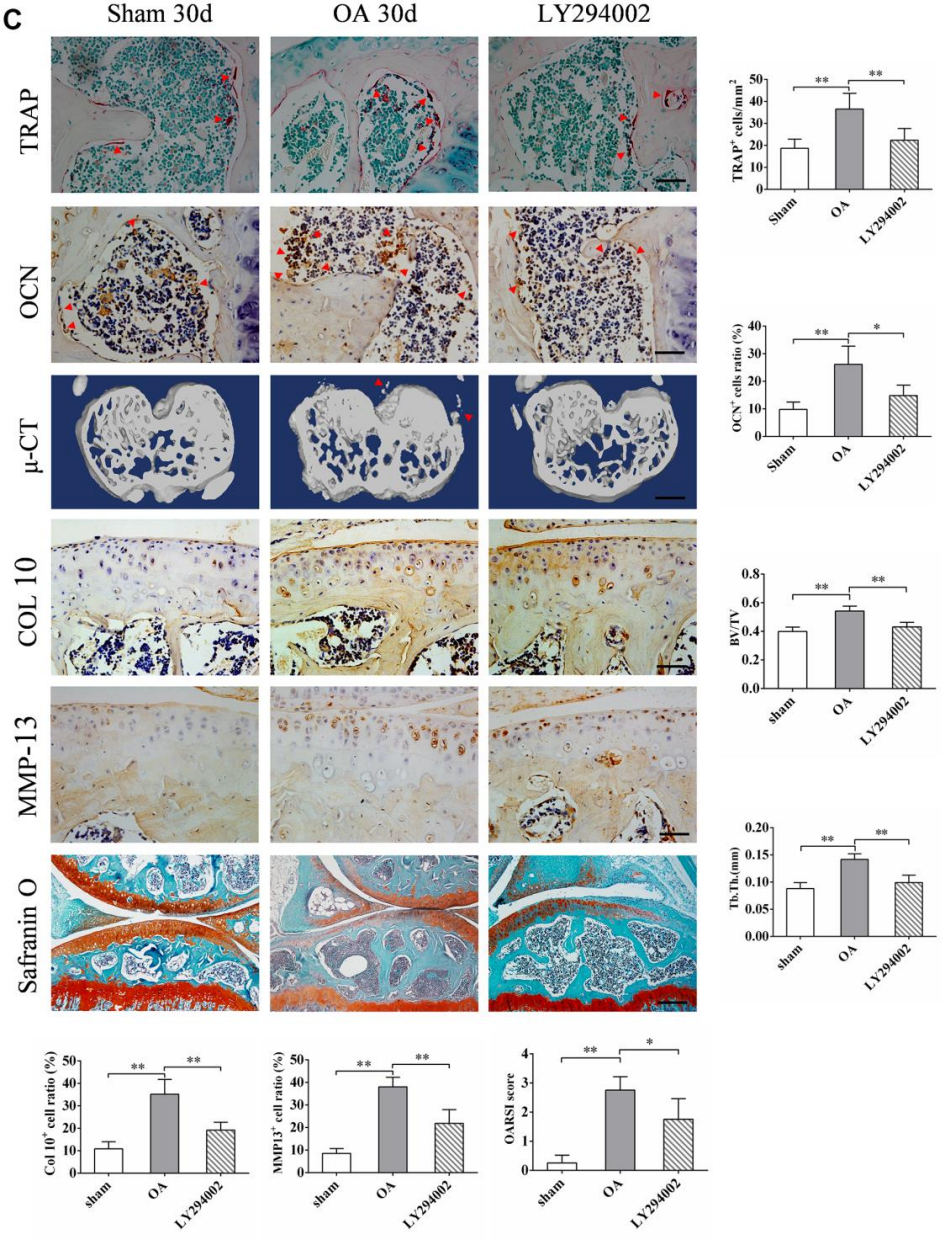
**LY294002 inhibits OPN-mediated subchondral bone and cartilage degeneration in OA.** (**C**) TRAP staining of osteoclasts and immunostaining of OCN+ cells were performed on subchondral bone of tibia in sham group, OA group and LY294002 treatment group. Positive cells were indicated with arrows, scale bars = 50 μm, n=6. Safranin O-fast green staining and OARSI scores of tibial articular cartilage in sham group, OA group and LY294002 treatment group. Scale bars = 200 μm. Representative 3D reconstruction of micro-CT images of tibial subchondral bone in sham group, OA group and LY294002 treatment group; scale bars = 1 mm. Quantitative analysis of bone volume/total volume (BV/TV) and trabecular thickness (Tb. Th.), n=6. Representative immunostaining and quantitative analysis of COL 10+, MMP-13+ cells in articular cartilage in sham group, OA group and LY294002 treatment group. Scale bars = 50 μm, n=6. Data are shown as mean ± s. d. and were analyzed by one-way ANOVA, *P < 0.05, **P < 0.01.

## DISCUSSION

In this present study, we found increased expression of OPN in subchondral bone of OA patients and OA mouse models induced by ACLT+DMM. The increased expression of OPN accelerated OA subchondral bone turnover and bone remodeling, promoted the formation of h-type vessels in subchondral bone, and mediated the damage of articular cartilage caused by subchondral bone metabolism. We also confirmed that the increased expression of OPN in OA subchondral bone mainly in preosteoblasts and osteoblasts. In addition, our results suggested that the increased expression of OPN in OA subchondral bone may be regulated by PI3K/ AKT signaling pathway. Inhibition of PI3K/ AKT signaling can inhibit OPN-mediated subchondral bone and cartilage changes in OA.

The remodeling of OA subchondral bone is very active, involving bone resorption and formation. Absorption and destruction of the trabecular bone can be observed in the subchondral bone of patients with early OA [26]. Aizah et al. found in an experimental animal model of OA that early subchondral bone trabeculae became thin and decreased, and bone mineral density and bone volume also decreased [27]. The activation and abnormal proliferation of osteoclasts are the key to the imbalance of bone resorption and bone metabolism. Another critical step in bone resorption is the adhesion of osteoclasts to a mineralized matrix on the bone surface. Previous studies have shown that OPN is highly expressed on the bone surface where osteoclasts anchor [28], and OPN-specific receptor-cell surface adhesion molecule is also preferentially expressed in the corresponding region of the osteoclast membrane [29]. These findings suggest that OPN plays an important role in activating osteoclast bone resorption. We found that injecting rmOPN into OA mouse model induced the formation of more osteoclasts in OA subchondral bone, while injecting OPN neutralizing antibody into OA mouse model reduced the number of osteoclasts in OA subchondral bone. The addition of exogenous rmOPN can induce BMMs to express more RANKL *in vitro* and promote BMMs to differentiate more osteoclasts *in vitro*. These results suggest that OPN is involved in the pathological process of regulating the abnormal increase of OA subchondral osteoclasts and the increase of bone resorption.

Changes in OA subchondral bone metabolism may be due to abnormal osteocyte activity, reflected in the formation of abnormal collagen or non-collagen components, or due to low mineralization of bone tissue [30]. Abnormal activity of osteoblasts may lead to poor mineralization of collagen matrix, increase mechanical pressure on articular cartilage, decrease cartilage repair ability, and result in degradation of cartilage matrix [31]. These metabolic changes were detected by measuring the metabolic biochemical indices of OA subchondral osteoblasts. Sanchez et al. isolated osteoblasts from the subchondral osteosclerosis area of OA patients for primary culture and examined the phenotype of osteoblasts. It was found that the expression levels of MMP-13, COL1A1, COL1A2, tissue nonspecific AP, VEGF, ANKH, TGase 2, XIIIA and dental matrix protein 1 genes, including OPN, were significantly up-regulated in sclerotic osteoblasts compared with non- sclerotic osteoblasts [32]. In addition, compared with normal cells, primary OA subchondral osteoblasts showed increased alkaline phosphatase activity and osteocalcin secretion under alkaline conditions and 1,25 (OH)2D3 stimulation [33]. It is suggested that the abnormal phenotype of subchondral osteoblasts is an important cause of OA subchondral osteosclerosis [34]. We found that MC3T3-E1 cells increased alkaline phosphatase activity and expression of COL1A, OCN and RUNX2 under the induction of Dex, β-glycerophosphate and VC and the addition of exogenous OPN. The expression of OPN was increased in preosteoblasts and osteoblasts of OA subchondral bone, which may promote the proliferation and differentiation of preosteoblasts and osteoblasts of subchondral bone, leading to changes in OA subchondral bone metabolism and subchondral bone sclerosis.

Angiogenesis is another important pathological feature of subchondral bone in osteoarthritis. It is associated with subchondral bone turnover and bone remodeling, especially the h-type vessels. H-type vessels are a newly discovered special subtype in recent years, and play an important role in promoting bone neoangiogenesis and bone mass increase [35]. Recent studies have reported increased expression of h-type vessels in the subchondral bone of OA mice [36], and the degree of cartilage degeneration in OA mice was significantly correlated with the number of h-type vessels [37]. In addition, in late OA, new blood vessels cross the bone-cartilage junction and grow to the uncalcified cartilage layer, leading to endochondral calcification and aggravating the degeneration of articular cartilage [38]. We found that injection of rmOPN in OA mice induced more h-type vessel formation in OA mouse subchondral bone, while injection of OPN neutralizing antibody in OA mice reduced the formation of h-type vessel in OA mouse subchondral bone. *In vitro* stimulation with exogenous rmOPN promoted the proliferation of HUVECs and induced HUVECs to form tubes. These results suggest that OPN is involved in the regulation of OA subchondral bone h-type angiogenesis, and h-type angiogenesis contributes to bone metabolism and bone remodeling, which may be another important mechanism by which OPN regulates OA subchondral bone metabolism and bone remodeling.

Altered subchondral bone and articular cartilage interactions have been implicated in the pathogenesis of OA [39, 40]. The secretory activity of osteoblasts in subchondral bone in OA patients promotes the degeneration of chondrocytes. Chondrocytes were isolated from articular cartilage and co-cultured with osteoblasts from OA subchondral bone or normal subchondral bone. It is found that OA subchondral osteoblasts blocked the expression of chondrocyte aggrecan, but promoted the expressions of disintegrins and metalloproteinases with thrombospondin motifs (ADAMTs) and matrix metalloproteinases (MMPs) [41]. These results suggest that subchondral osteoblasts in OA patients induce cartilage degradation by stimulating chondrocytes to produce ADAMTs and MMPs with motifs of thrombocyte reactive protein, and inhibit the synthesis of aggregated proteoglycan. In Sanchez et al. 's study, they also found that subchondral osteoblasts in OA patients showed a down-regulating effect on the expression of type 2 collagen in human chondrocytes [42], suggesting that subchondral osteoblasts in OA patients may cause hypertrophic differentiation of chondrocytes, and cause matrix mineralization, finally leading to cartilage degeneration. Increased expression of OPN in subchondral bone in OA may promote cartilage catabolism and degeneration. We found that after the induction of chondrogenic differentiation of ADTC5 cells *in vitro*, the addition of exogenous rmOPN inhibited the synthesis of proteoglycan in the extracellular matrix of chondrocytes and promoted the hypertrophic differentiation of chondrocytes. We also found that OPN injection in OA mice can promote the expression of collagen 10 and MMP13, accelerate the degeneration of articular cartilage, and the injection of OPN neutralizing antibody can alleviate the degeneration of articular cartilage in OA mice.

Subchondral osteosclerosis plays a crucial role in the occurrence and development of OA [43]. Although the role of subchondral bone in the pathogenesis of OA is not fully understood, the close anatomical relationship between subchondral bone and articular cartilage determines its special role. The results of this study indicate that OPN plays an important role in the pathological changes of OA subchondral bone. Increased expression of OPN accelerates bone turnover and bone remodeling of OA subchondral bone, promotes vascular proliferation in OA subchondral bone, and mediates degeneration of articular cartilage caused by changes in OA subchondral bone metabolism. However, the specific reasons for the up-regulation of OPN expression in subchondral bone of OA are still unclear, and may be related to the increase of mechanical stress on the articular surface due to joint degeneration. In addition, the limitation of this study is that we used neutralizing antibodies against OPN rather than achieving subchondral bone-specific knockout of OPN to verify the effect of OPN on subchondral bone and cartilage, which will be improved in future studies. To clarify the mechanism of OPN expression in subchondral bone in OA is of great value and significance for exploring the specific biological indicators for the early diagnosis and monitoring of OA disease progression, developing drugs to regulate the metabolic activity and intensity of bone remodeling of subchondral bone, and improving subchondral sclerosis.

## MATERIALS AND METHODS

### Human subchondral bone tissue

OA subchondral bone tissue was obtained from OA patients undergoing total knee replacement (n = 16). Normal subchondral bone tissue was obtained from traumatic amputation patients with no evidence of cartilage degeneration (n = 5). Both normal and OA bone fragments were collected from the middle of the septum medial to the tibial plateau of the knee bearing. Bone tissue samples were taken for protein extraction, hematoxylin and eosin (H&E) staining, and histological analysis. All human samples were collected from Shantou Central Hospital (Shantou, China), and patients agreed to the use of their clinical information for scientific research. This study was approved by the Ethics Committee of Shantou Central Hospital.

### Animals, destabilization-induced osteoarthritis model and treatment

Male C57BL/6J mice at 12 weeks of age were purchased from Guangdong Experimental Animal Center (Guangzhou, China). Animals were housed in groups of five mice per cage in a specific-pathogen-free (SPF) room at 20 to 22° C under a 12/12 h light/dark cycle with 50 to 60% humidity and provided filtrated water and chow ad libitum. To establish the OA model, mice were randomly allocated to either (1) sham group or (2) OA group, with 12 mice in each group. In sham group, mice’ knees underwent only a skin and capsule incision. In OA group, the knees of the mice were subjected to anterior cruciate ligament transection and destabilization of the medial meniscus (ACLT + DMM) according to previous methods [44]. Mice were anesthetized with 300 mg/kg intra-peritoneal Tribromoethanol (Aldrich, Milwaukee, WI) and knees were prepared for aseptic surgery. Buprenorphine (Buprenex®, Reckitt and Coleman Products, Kingston-Upon-Hull, UK) was provided peri-operatively at 0.09 mg/kg. Surgical equipment included a surgical microscope (Leica LZ-6, Leica Microsystems Inc., Bannockburn, IL); Sharpoint® 15° 5 mm blade micro-surgical knife; Absorption Spears (Fine Science Tools Inc., Foster City, CA); micro-iris scissors; micro-surgery needle holders; micro-corneal suturing forceps; Jewelers forceps and # 15 blades. Six mice were sacrificed 30 days or 60 days after surgery, and the knee joint samples were removed for cell, total RNA and protein extraction, and histological analysis.

For OPN or antibody treatment *in vivo*, mice were randomly allocated to one of the following groups: (1) sham group, (2) OA+vehicle group, (3) OA+OPN group and (4) OA+antibody group, with 12 mice in each group. RmOPN (200 μg/mouse; R&D Systems, # 441-OP) or anti-OPN antibody (50 μg/mouse, R&D Systems, # AF808) was injected intraperitoneally into C57BL/6J mice twice a week for 4 or 8 weeks as previously described [45] and according to the manufacturer’s instructions. The control groups were all treated with saline for the same periods. Six mice were sacrificed 30 days or 60 days after surgery.

For LY294002 treatment *in vivo*, mice were randomly allocated into (1) sham group, (2) OA group, or (3) OA+LY294002 group, with six mice in each group. LY294002 (350 mg/kg, Sigma-Aldrich) was injected intraperitoneally into C57BL/6J mice twice a week. The mice were sacrificed 30 days after surgery. All animal experiments were approved by the Ethics Committee of Shantou Central Hospital. and were performed in accordance with the Committee’s guidelines.

### Cell preparation

Mouse preosteoblast cells (MC3T3-E1), human umbilical vein endothelial cells (HUVECs), and mouse chondroprogenitor cells (ATDC5) were purchased from the Cell Bank of the Type Culture Collection of the Chinese Academy of Sciences (Shanghai, China). Primary osteoblasts from subchondral bone were obtained and cultured as our previously described [25]. To initiate differentiation, MC3T3-E1 cells were treated with Dex (10^−7^ M), β-glycerophosphate (10 mM) and vitamin C (VC, 50 μg/mL) for 3 or 5 days to stimulate osteogenesis. ADTC5 cells were cultured in Dulbecco's Modified Eagle Medium (DMEM) and Ham's F12 (DMEM) 1:1 mixed Medium containing 5% fetal bovine serum (Gibco) and treated with insulin-transferrin-selenium (ITS, 10 μg/mL; Sigma-Aldrich, St Louis, MO, USA) for cartilage differentiation [46]. Bone marrow-derived macrophages (BMMs) were obtained and cultured as previously described [47]. Briefly, bone marrow was washed out from the femora and tibiae and centrifuged at 500 × g for 10 min. Cells were resuspended and cultured in α-MEM supplemented with 10% FBS, 1% penicillin/streptomycin in a 37° C, 5% CO2 incubator overnight. Then the non-adherent cells were seeded into α-MEM supplemented with macrophage colony-stimulating factor (M-CSF; 20 ng/mL; PeproTech). After 48 h, the non-adherent cells were discarded and adherent cells were used as BMMs. BMMs were treated with recombinant murine soluble RANKL (50 ng/mL; PeproTech, Rocky Hill, NJ, USA) and M-CSF (20 ng/mL; PeproTech) for 5 or 7 days to stimulate osteoclastogenesis, and the differentiation medium was replaced every 2 days. BMMs, HUVECs, ADTC5 and MC3T3-E1 cells were treated with 100 ng /mL rmOPN (R&D Systems, Minneapolis, MN, USA, # 441-OP) and 1.0 μg/mL anti-OPN antibody (23C3, R&D Systems, # AF808) as previously described [48, 49].

### siRNA interference

PTEN siRNAs (sense 5′-GCA CAA GAG GCC CUA GAU UTT-3′; antisense 5′-AAU CUA GGG CCU CUU GUG CTT-3′) and negative scrambled control siRNA were purchased from Genepharma Co., Ltd. (Shanghai, China). According to the protocol provided by the manufacturer, MC3T3-E1 cells were transfected with PTEN siRNA or FAM negative siRNA in optimized Eagle medium (opti-MEM) (Invitrogen, Carlsbad, CA, USA) using Lipofectamine® 2000 (Invitrogen). After transfection, MC3T3-E1 cells were incubated at 37° C for 6 h, then the transfection medium was replaced with α-MEM supplemented with 20% FBS. The cells were further incubated for 48 h. Untransfected cells were used as negative controls. The transfected cells were treated with LY294002 (10 μM, Sigma-Aldrich) for 48 h, and total protein were extracted from the cells.

### Real-time PCR gene expression analyses

Total RNA was isolated from cell pellets (1 ×10^6^ per well in 12 wells plate) using TRIzol reagent (Life Technologies, Grand Island, NY, USA), cDNA was made from RNA samples (1000 ng per sample) using reverse transcription reagents (Vazyme Biotech Co. Ltd, Nanjing, China) and quantitative PCR assays were carried out to quantify mRNA expression levels of OPN, using a Real-Time PCR Mix (Vazyme azymeime PCR Mix in a Light Cycler (Roche Molecular Biochemicals, Indianapolis, IN, USA), with GAPDH gene used as the internal loading control. The following primer sequences were used: OPN (forward primer: 5′-GTGGGAAGGACAGTTATCAA-3′; reverse primer: 5′-CTGACTTTGGAAAGTTCCTG-3′); GAPDH (forward primer: 5′-CAATGACCCCTTCATTGACC -3; reverse primer, 5′-GACAAGCTTCCCGTTCTCAG -3′).

### Western blotting

After treatment, the cells (1 ×10^6^ per well in 12 wells plate) were lysed immediately for 5 min at 95° C in buffer (62.5 mM Tris HCl, pH 6.8, 10% glycerol, 2% sodium dodecyl sulfate, 50 mM dithiothreitol and 0.01% bromophenol blue). Cell lysates (30ug protein amount per group) were analyzed by SDS-PAGE and transferred to a nitrocellulose membrane (Millipore, Billerica, MA, USA). Blots were probed with primary antibodies against AKT (Santa Cruz Biotechnology, CA, USA; sc-55523), phosphorylated (p)-AKT (Santa Cruz Biotechnology, sc-293125), osteopontin (OPN, Proteintech, Rosemont, IL, USA; 22952-1-AP), PTEN (Abcam, ab170941), RANKL (Proteintech, 23408-1-AP), collagen 1a (COL1a, Abcam, Cambridge, MA, USA; ab255809), RUNX2 (Immunoway, Plano, TX, USA; YT4192), osteocalcin (Santa Cruz Biotechnology, sc-390877), MMP-13 (Affinity Biosciences Ltd., Cincinnati, OH, USA; AF5355), collagen 10 (Abcam, ab182563), β-actin (Proteintech, 20536-1-AP), and immunoreactive proteins were revealed using an enhanced chemiluminescence kit (Santa Cruz Biotechnology). Western blot data were evaluated as follows: the gray values of the western blot bands were quantified by Image-ProPlus 4.5 software (Media Cybernetics, Rockville, MD) in the control and experimental groups. As an estimate of the protein level, the gray value of the protein of interest of each group was normalized with (divided by) the gray value of internal reference protein.

### ALP staining and ALP activity assay

MC3T3-E1 cells were seeded into 24-well plates (8 × 10^4^ cells/well), and treated with 10^−7^ M Dex, 10 mM β-glycerophosphate, 50 μg/mL vitamin C (VC) and 100 ng/mL rmOPN and 1.0 μg/mL anti-OPN antibody for 3 or 5 days to stimulate osteogenesis, then washed with PBS. ALP staining was performed according to the manufacturer's instructions (Sigma-Aldrich). Alkaline phosphatase activity was assessed using the alkaline phosphatase detection kit and evaluated according to the manufacturer's instructions (Beyotime, P0321). ALP activity was normalized to the total intracellular protein content and presented as mU/mg protein.

### Toluidine blue staining

ADTC5 cells were seeded into 6-well plates (4 × 10^5^ cells/well), and treated with insulin-transferrin-selenium (ITS, 10 μg/mL; Sigma-Aldrich) for chondrogenic differentiation, and treated with 100 ng/mL rmOPN and 1.0 μg/mL anti-OPN antibody for 14 days. Cultured cells were fixed with 4% paraformaldehyde for 30 minutes at room temperature, then stained using a toluidine blue staining kit (Leagene, Beijing, China) for 60 minutes at room temperature, and washed with PBS to remove excess dye.

### Proliferation assay

HUVECs were plated into a 12 well plate at a density of 1 × 10^5^ cells/well. After 24 hours treatment with 100 ng/mL rmOPN and 1.0 μg/mL anti-OPN antibody, cells were incubated with 100 μg/mL BrdU for 2 h according to the protocol (MedChemExpress, Monmouth, NJ, USA, HY-15910). For quantification, BrdU-positive cells were counted in three random fields. Images were captured using a fluorescence microscope (Olympus, Tokyo, Japan).

### Tube formation assay

After treated with 100 ng/mL rmOPN and 1.0 μg/mL anti-OPN antibody for 24 h, HUVECs were coated with 200 μL of matrigel in 24-well dishes at a cell density of 1 × 10^4^ cells/well. Tube formation was assessed by visual microscopy with an inverted microscope (Olympus). The parameter of tube formation were analyzed using the angiogenesis analysis plugin of the Image J software (NIH, Bethesda, MD, USA).

### Micro-CT imaging

After fixed in 4% paraformaldehyde for 24 h, micro-CT scanning was performed of the mouse knee joints at 100 kV, 98 μA, 12 μm resolution on a micro-CT Scanner (Viva CT40, ScancoMedical AG, Bassersdorf, Switzerland). The tibial subchondral bones were chosen as the region of interest for three-dimensional (3D) reconstruction and morphometric parameter analyses. The 3D morphometric parameters analyzed included bone volume/tissue volume (BV/TV) and trabecular thickness (Tb. Th.).

### Hematoxylin and eosin (H&E), safranin o-fast green, TRAP staining, immunohistochemical and immunofluorescence staining

The knee joints were removed from the surrounding muscles and fixed in 4% paraformaldehyde for 24 h. Whole joints were decalcified in 10% EDTA (pH 7.4) at 4° C for 30 days on a shaker. Joints were embedded in paraffin and 4 μm sagittal sections were taken through the entire joint at 80 μm intervals. H&E and safranin O-fast green staining were used to assess the severity of cartilage degeneration according to the OARSI system, as described previously [50]. Tartrate-resistant acid phosphatase (TRAP) staining was performed according to the manufacturer’s instructions (Sigma-Aldrich). Immunostaining was performed according to the manufacturer’s instructions. We incubated sections with primary antibodies against Osterix (Santa Cruz Biotechnology, sc-393060), OPN (Proteintech, 22952-1-AP), β-tubulin (Proteintech, 10094-1-AP), OCN (Santa Cruz Biotechnology, sc-390877), CD31 (R&D Systems, AF3628), Endomucin (EMCN, Santa Cruz Biotechnology, sc-65495), MMP-13 (Affinity Biosciences Ltd., AF5355), collagen 10 (ABclonal, A6889), and p-AKT (Cell Signaling Technology, #4060) overnight at 4° C. For immunofluorescent staining, secondary antibodies conjugated with fluorescent tags were added and slides were incubated at room temperature for 1 h in the dark. The number of positively-stained cells in the tibia subchondral bone area was counted in three sequential sections per mouse in each group.

### Statistical analyses

All experiments were performed in duplicate or triplicate and observed by independent observers. Differences between two groups were statistically analyzed by unpaired, two-tailed Student’s t-test. Differences among three groups were analyzed by one-way analysis of variance (ANOVA) and Tukey’s multiple comparison test. All statistical analyses were performed with GraphPad Prism software version 6.0 (GraphPad Software, Inc., La Jolla, CA, USA). The results are presented as the mean ± standard deviation (s. d.), and *P* < 0.05 was considered statistically significant.

### Ethics statement

The studies involving human participants were reviewed and approved by the Ethics Committee of Shantou Central Hospital, Shantou Hospital, China. The patients/participants provided their written informed consent to participate in this study. The animal study was reviewed and approved by the Ethics Committee of Shantou Central Hospital, Shantou Hospital, Sun Yat-sen University, China.
